# Mitochondrial Phylogenomics of Scoliidae from China, with Evidence to Challenge the Former Placement of the *Colpa* Group

**DOI:** 10.3390/insects15100758

**Published:** 2024-09-30

**Authors:** Zhen Liu, Cornelis van Achterberg, Huayan Chen

**Affiliations:** 1 Zoology Key Laboratory of Hunan Higher Education, College of Life and Environmental Sciences, Hunan University of Arts and Science, Changde 415000, China; qingniao8.27@163.com; 2 State Key Laboratory of Development Biology of Freshwater Fish Sub-Center for Health Aquaculture, Hunan University of Arts and Science, Changde 415000, China; 3 Science and Technology Innovation Team for Efficient Agricultural Production and Deep Processing at General University in Hunan Province, Hunan University of Arts and Science, Changde 415000, China; 4 Institute of Insect Sciences, Zhejiang University, Hangzhou 310058, China; kees@vanachterberg.org; 5 Guangdong Provincial Key Laboratory of Applied Botany, South China Botanical Garden, Chinese Academy of Sciences, Guangzhou 510650, China; 6 State Key Laboratory of Plant Diversity and Specialty Crops, South China Botanical Garden, Chinese Academy of Sciences, Guangzhou 510650, China; 7 South China National Botanical Garden, Guangzhou 510650, China

**Keywords:** mitogenome, gene arrangement, phylogeny, classification

## Abstract

**Simple Summary:**

Little is known about the molecular data and phylogenetic relationships of scoliid wasps. In this study, we determined 10 mitochondrial genomes representing eight genera in two tribes of the family Scoliidae distributed in China, compared and analyzed all Chinese genus representatives for general features and rearrangement. Phylogenetic relationships between them were inferred using MrBayes and IQtree based on four data matrices. Gene rearrangement was chosen for ancestral state reconstruction. Our results support most of the relationships between genera and tribes of Scoliidae in former morphological studies except for the *Colpa* group, which is proved to be closer to Scoliini based on our genome features, phylogenetic analyses and some morphological evidence.

**Abstract:**

Scoliidae, also known as scarab hunters or flower wasps, are important in the biological control of scarabs and for pollination. Mitogenomic and phylogenetic studies are rare for this group. In this study, 10 mitochondrial genomes representing eight genera in two tribes of the family Scoliidae were determined. The general features and rearrangements of the mitochondrial genomes for 15 Scoliidae species representing all genera distributed in China were described and compared and the phylogenetic relationships among them were inferred using MrBayes and IQtree based on four data matrices. Most sequences of Scoliidae have one extra *trn*M gene. Species belonging to Campsomerini have lower A + T content than all Scoliini species except for *Colpa tartara* in this study. The AT-skew is positive in 7 out of 15 species. All 15 Scoliidae sequences have similar conserved gene arrangements with the same arrangements of PCGs and rRNA genes, except for *Campsomeriella annulata*. The tRNA genes have the highest frequency of rearrangement, and *C. tartara* is always rearranged as in its Scoliini counterparts. Our phylogenetic results support most of the relationships between genera and tribes of Scoliidae in former morphological studies. However, *Colpa tartara* is proved to be closer to Scoliini according to genome features, phylogenetic analyses and some morphological evidence, which challenges the former attribution of the *Colpa* group.

## 1. Introduction

Scoliid wasps (Hymenoptera, Scoliidae), also known as scarab hunters or flower wasps, are pollinators for various plants and larval parasitoids of scarabaeoid beetles [1]. These wasps comprise approximately 560 extant and 19 extinct species worldwide [2,3]. This family traditionally had been grouped with many Tiphiidae, particularly Anthoboscinae in a single superfamily Scolioidea for sharing a general habitus as solitary fossorial aculeate wasps [4]. Brothers [5] (as Königsmann [6]) reevaluated the relationships of the Aculeata and combined the traditional superfamilies Scolioidea, Pompiloidea, Vespoidea and Formicoidea into the superfamily Vespoidea, since these formed a monophyletic clade. The family Scoliidae was also incorporated into Vespoidea later in the phylogenetic analysis of morphological data by Brothers [7]. Phylogenetic relationships among major lineages of the Aculeata have been mainly studied in the 21st century [8,9,10,11]. The Scoliidae was elevated to superfamily level inside the clade Aculeata based on morphological and molecular data by Pilgrim et al. [9]; this was also supported by Branstetter et al. [8], Peters et al. [12] and Blaimer et al. [13], based on transcriptomes and ultraconserved elements (UCE), as well as Zheng et al. [11] and Huang et al. [14], based on mitochondrial genomes.

Scoliid wasps are difficult to determine, as suggested by the description of many subspecies and varieties and shown by their extreme sexual dimorphism. Phylogenetic research on this group has been rare: only a few scattered notes were published during the last century. All scoliid species were classified into Scoliinae and Campsomerinae [15,16] before a new subfamily Proscoliinae was found and the rest were subjected to the subfamily Scoliinae with two tribes, Scoliini and Campsomerini, designated by Rasnitsyn in 1977 [17]. The hierarchical levels were supported by Brothers [7] and adopted by many researchers [1,2,18,19,20,21,22,23,24,25]. Later, Rasnitsyn [26] added an extinct subfamily, Archaeoscoliinae. Argaman [27] divided the family into 143 genera and four subfamilies (Proscoliinae, Colpinae, Scoliinae and Campsomerinae) but his arrangement was not followed by most taxonomists because of insufficient diagnoses without taxonomic revisions. For instance, Osten [2] deliberately ignored it and listed only 43 valid genera. Nevertheless, Argaman discovered some valuable characters for morphological taxonomy and recognised the placement problem of the Colpini or Trielidini (here, we use the latter based on Betrem and Bradley [16], according to the ICZN Code) species. Moreover, the campsomerine species were suggested to be elevated to the subfamily level by Cornelis van Achterberg for their extreme sexual dimorphism and distinct discrepancy in body size of both sexes compared to other groups. No molecular study had attempted to reconstruct a phylogeny of the family before Khouri et al. [28] made the first attempt using UCE data. They confirmed the position of *Proscolia* as the sister to all other extant scoliids and supported the sister group relationship between *Colpa* and the Scoliini in their preprint (not formally published), with an emphasis on a dire need for revision of the higher-level taxonomy of the Scoliidae.

In China, only the subfamily Scoliinae (including the Campsomerini) has been reported and it comprises 11 genera with 61 known species currently [24,25,29,30]. The phylogenetic tree [1] based on the *COI* sequences of 22 morphospecies belonging to nine genera in two tribes of the subfamily Scoliinae from Southern China corroborates the tribal system of Day et al. [4], indicating that mitochondrial sequences are phylogenetically informative for addressing the relationships within Scoliidae. However, the relationships among extant genera of the family are far from clear. For example, Trielidini species were not included in the above study. Recent comparative studies of the mitochondrial genomes of the Aculeata show that both gene arrangements and gene sequences provide insights into the phylogeny of the group [11,14,31,32,33]. Compared to other groups of Hymenoptera, however, there is a serious lack of representative mitochondrial genomes of Scoliidae, which is an obstacle to the study of phylogenetic relationships within Scoliidae.

Here, we successfully collected, sequenced and described 10 mitochondrial genomes of Scoliidae using next-generation sequencing. We also compared genome features of 15 Scoliidae species covering all 11 genera known from China and we analyzed phylogenetic relationships and their influences to illuminate evolutionary patterns across Scoliidae.

## 2. Material and Methods

### 2.1. Collection, Identification and DNA Extraction

Specimens were collected by hand netting or Malaise traps (MTs) set up in Guangdong, Yunnan, Hainan and Shaanxi except for one specimen of *Campsomeriella annulata* (Fabricius, 1793) from Bhutan (Table S1). The collected specimens were stored in 100% ethanol and at −80 °C prior to DNA extraction.

Genomic DNA was extracted from the legs of specimens using an Ezup Column Animal Genomic DNA Purification Kit (Sangon Biotech, Shanghai, China) following the manufacturer’s protocols. Extracted genomic DNA was quantified by NanoPhotometer^®^ (IMPLEN, Westlake Village, CA, USA) and Qubit 3.0 (Invitrogen, Life Technologies, Carlsbad, CA, USA) and a Nanodrop 2000c Spectrophotometer (Thermo Scientific, Wilmington, DE, USA). Voucher specimens are deposited in the Laboratory of Hunan University of Arts and Science, Changde, China.

### 2.2. Genome Sequencing, Assembly, Annotation

The extracted genomic DNA of each specimen was sheared into fragments of approximately 350 bp in length using an Ultrasonic Processor Covaris S220 (Covaris, Inc., Woburn, MS, USA). High-throughput sequencing libraries were constructed using an Illumina TruSeq DNA PCR-Free HT Kit and sequenced using an Illumina Novaseq6000 with the strategy of producing 150 bp paired-ends by the Annoroad Gene Tech. (Beijing, China) Co., Ltd. The quality of raw sequencing reads was checked by a FastQC version 0.11.3 [34], and low-quality reads and sites were filtered by a Trimmomatic version 3.2.57 [35].

The target mitochondrial reads were filtered out using BLAST (BLASTn with an E value: 1 × 10^−5^) against a reference data set containing Hymenoptera mitochondrial genomes via the FastqExtract script [36]. The mitochondrial genomes were assembled by IDBA_UD version 1.1.3 [37] and SPAdes version 3.15.2 [38] with default parameters.

Annotation of the assembled genomes was performed initially by using MITOS Web Server [39]. Start and stop codons of protein-coding genes (PCGs) were manually adjusted in Geneious Prime v11 by referring to the published mitogenomes of Aculeata. All mitochondrial genomes were submitted to GenBank under the accession numbers PP874258−PP874267.

### 2.3. Genome Feature and Arrangement Analysis

The base compositions and codon usages (RSCU) in each sequence were calculated according to methods described in Yuan et al. [40]. AT-skew and GC-skew were calculated as AT-skew = (A − T)/(A + T) and GC-skew = (G − C)/(G + C) [41]. Gene arrangements of mitochondrial genomes in Scoliidae were compared using 17 representative mitochondrial genomes (Table 1).

### 2.4. Phylogenetic Inference

A total of 15 species covering all 11 genera traditionally or morphologically accepted Chinese scoliids were used in phylogenetic analyses. Two species representing two families of Ichneumonoidae were chosen as outgroups (Table 1). The nucleotide sequences of 13 protein-coding genes (PCGs) were aligned using G-INS-i algorithms implemented in MAFFT v7.407 [44].

The ambiguously aligned positions were identified and removed by the combination of Aliscore version 2.2 and Alicut version 3.2 to reduce noise [45]. The best partition schemes of the substitution models for the matrix were searched in PartitionFinder v2.1.12 with model selection = BIC and Branch lengths = unlinked between different subsets [46].

Four data matrices for phylogenetic inferences, i.e., DNA sequences of all 1st, 2nd and 3rd positions of codons in protein-coding genes (PCG123), all 1st and 2nd protein-coding genes (PCG12), DNA sequences of all protein-coding and RNA genes (PCG + RNA) and amino acid sequences of all protein-coding genes (AA).

Bayesian inference (BI) analyses in MrBayes v3.2.7a and Maximum Likelihood (ML) analyses in IQtree v1.6.12 [47,48] were used to reconstruct the phylogenetic relationship. In MrBayes analysis, substitution models were applied for each partition chosen by PartitionFinder. Four independent Markov chains were run for 10 million generations, with tree sampling occurring every 1000 generations and a burn-in of 25% of trees. The stationarity of the run was checked by the program Tracer version 1.4.0 (effective sample sizes > 200). In ML analysis, 200 runs were conducted to find the highest-likelihood tree, followed by analysis of 1000 bootstrap replicates.

### 2.5. Phylogenetic Hypothesis Tests

We examined three conflicting nodes by four-clusters likelihood mapping analysis in TREE-PUZZLE version 5.2 [49]. All four matrices were used for the hypothesis testing. The TVM + F + I + G4 model was applied for PCG123 and PCG12, the GTR + F + I + G4 was for PCG + RNA and the mtZOA + F + R4 was for AA.

Gene rearrangement clusters (1 and 2 with two modes each) were chosen for ancestral state reconstruction. The data about rearrangement of clusters in Scoliidae mitogenomes were mapped on the phylogenetic tree (PCG123 + RNA) using Mesquite v3.81 [50]. The lack of data about the clusters was coded as missing data. We applied maximum parsimony and maximum likelihood reconstruction methods. Depending on the AIC criterion, we used the better fit model for the data: either the Mk1 model (Markov k-state 1parameter model) or the AsymmMk model (asymmetrical Markov k-state 2 parameter model).

## 3. Results

### 3.1. General Feature of the Mitochondrial Genomes in Scoliidae

**Figure 1:**
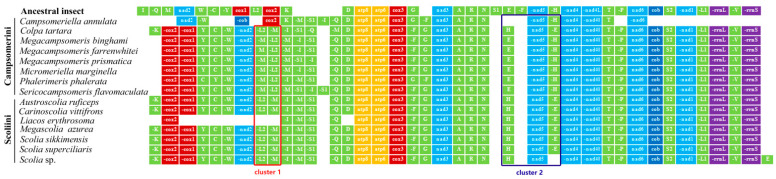
Gene rearrangement in 15 mitochondrial genomes of Scoliidae. Green stands for trnA genes, red stands for Cyclooxygenase genes, light blue stands for NADH dehydrogenase subunits genes, dark blue stands for cytochrome b gene, orange stands for ATP Synthase Membrane Subunits, and purple stands for ribosomal RNA genes. Two clusters (red box for cluster 1 and blue box for cluster 2) are selected for gene rearrangement analysis.

We sequenced mitochondrial genomes of 10 species from Scoliidae, representing eight genera in two tribes from more than 3 Gb of data for each specimen, covering 73% of the scoliid wasp genera distributed in China. Mitochondrial genomes were first reported for seven genera here, viz., *Austroscolia*, *Campsomeriella*, *Carinoscolia*, *Liacos*, *Micromeriella*, *Phalerimeris* and *Sericocampsomeris*. Ten nearly complete mitogenomes ranged in size from 15,183 bp in *Sericocampsomeris flavomaculata* to 16,863 bp in *Austroscolia ruficeps*, except for the shortly assembled *Campsomeriella annulata* and *Liacos erythrosoma* with 11,035 bp and 12,273 bp length, respectively. All protein-coding and rRNA genes were obtained except for *cox1* and *nad1* in *Campsomeriella annulata*, *cox1*, *cox2* and *nad2* in *Liacos erythrosoma* (Figure 1). Unlike most animal mitochondrial genomes, however, they all (except for the shortly assembled *C. annulata* and *L. erythrosoma*) have one extra *trnM* gene, just like the three sequences of *Megacampsomeris* published in Huang et al. [14].

**Figure 2:**
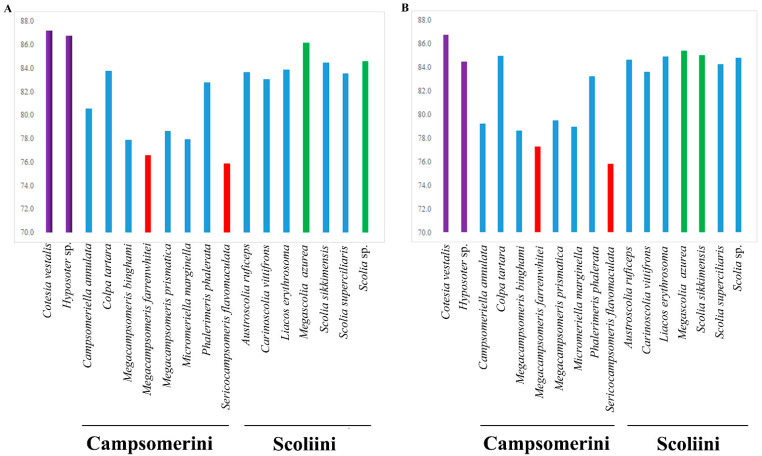
Base composition of the mitochondrial genomes of the Scoliidae and their hymenopteran outgroups. (**A**), A + T content for the sequenced region of the mitochondrial genomes in 17 species; (**B**), A + T content for protein-coding genes in 17 species. Red indicates the two lowest A + T content species. Green marks the two highest A + T content species (except the outgroups). Purple stands for outgroups. Blue is for other species.

**Figure 3:**
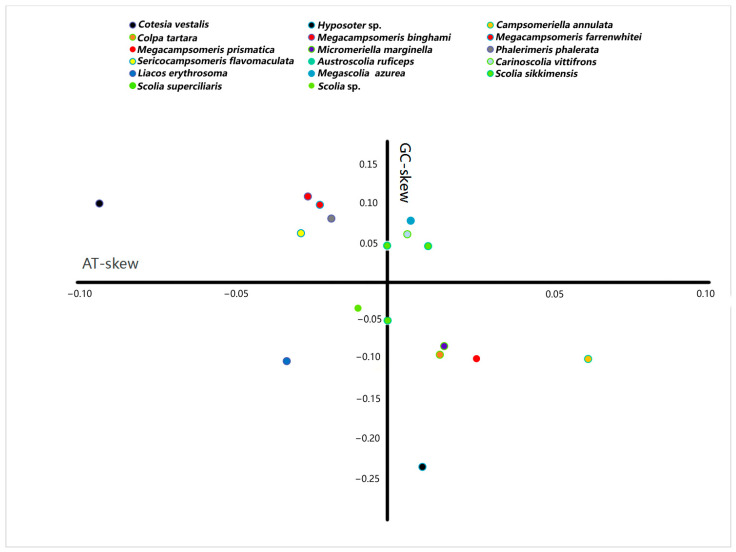
Strand asymmetry of the Scoliidae and the outgroups calculated from the majority strand of the mitochondrial genomes. Each mitochondrial genome is indicated by a filled circle. Different colors represent different genera of the Scoliidae and outgroups.

The A + T content for the sequenced region of the mitochondrial genomes in Scoliidae ranged from 75.9% (*Sericocampsomeris flavomaculata*) to 86.2% (*Megascolia azurea*). The lowest A + T content was found in the Campsomerini species, while the highest A + T content was found in the Scoliini species. Moreover, when compared with other available mitochondrial genomes from NCBI, all Campsomerini species had a lower A + T content than all Scoliini species in this study except for *Colpa tartara*, the only representative of Trielidini in China, with a relatively higher value (83.8%) than other Campsomerini representatives (Figure 2A). The A + T content was lower in the Scoliini (81.6%) than in the two outgroup species (87%) from the “Parasitica” group, which corresponded with the other Aculeata species in Zheng et al. [11]. The results were similar to 13 protein-coding genes (Figure 2B). In the Scoliidae, the AT-skew is positive in 7 out of 15 species while the GC-skew is negative in 7 out of 15 species (Figure 3), which is common in the Aculeata (see Zheng et al. [11]). However, AT-skew is positive and the GC-skew is negative in most species of insects [51].

**Figure 4:**
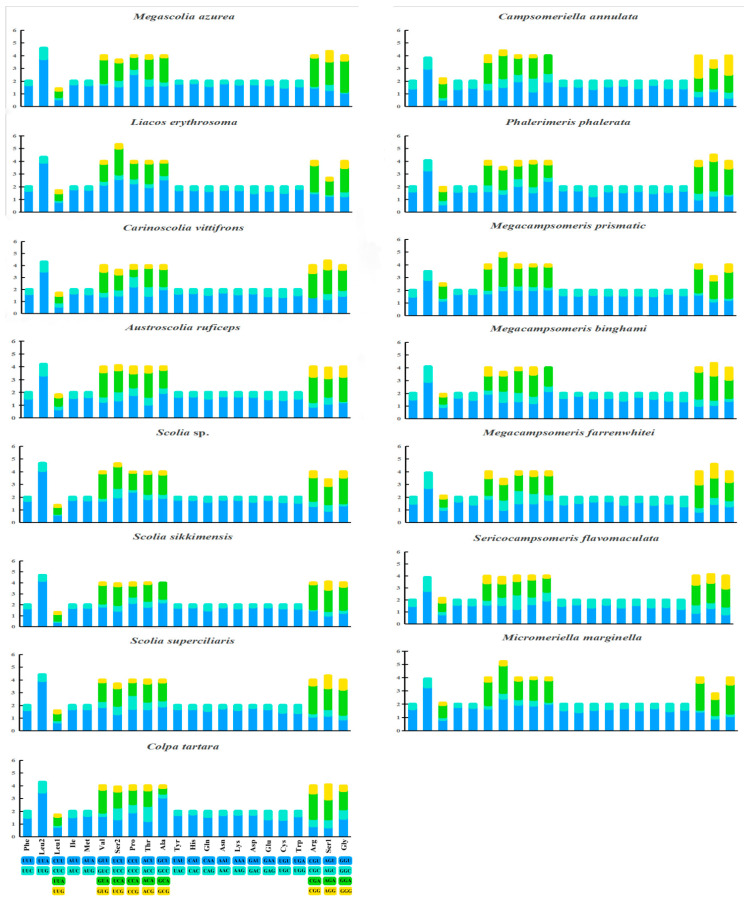
Relative synonymous codon usage (RSCU) of protein coding genes (PCGs) in 15 (10 new sequenced and 5 from NCBI) mitochondrial genomes of Scoliidae.

The relative synonymous codon usages (RSCUs) are shown in Figure 4. The PCGs, except for the stop codons, consist of 3412 codons in *Campsomeriella annulata* to 5241 codons in *Micromeriella marginella*. Seven AT-rich codons: AUU Ile (8.7%), AAU Asn (8.2%), AAA Lys (8.0%), UUU Phe (8.0%), UUA Leu (7.9%), AUA Met (7.1%), and UAU Tyr (6.8%), are the most frequent codons, which corresponds to the higher AT content in mitogenomes.

### 3.2. Gene Rearrangement in Scoliidae

Generally, in Scoliidae gene rearrangements were evident when compared with the ancestral type of the *Drosophila melanogaster* (Figure 1). Obviously, shuffled clusters between *trnI* to *trnK* and inverse transpositions in *trnF*, *trnS1*, and sometimes *trnE* occurred in all 15 Scoliidae sequences.

Overall, all 15 Scoliidae sequences had similar conserved gene arrangements with the same arrangements of PCGs and rRNA genes, except for the shortly assembled *C. annulata* which had a distinct inverse transposition of *cob* and shuffled cluster between *cox2* and *nad2*.

However, the tRNA genes had the highest frequency of rearrangement followed by PCGs in Scoliidae. The tRNA genes located in the tRNA clusters had a relatively higher frequency of rearrangement, such as the cluster between *nad2* and *trnD.* The *trnE*, *trnH* and *trnF* genes showed the most frequent transposition or inverse transpositions elsewhere. Other tRNA (*trnD*, *trnA*, *trnR*, *trnN*, *trnT*, *trnP*, *trnS2*, *trnL1* and *trnV*) genes without rearrangement were the most conservative.

Two obvious clusters can be observed to be different when comparing the two tribes: cluster 1 (red box in Figure 1) and cluster 2 (blue box in Figure 1). Scoliini members rearranged as “-*trnL2*-*(*-*trnM)*” (mode 1) for cluster 1 and “*trnH-nad5-(-trnE)*” (mode 3) for cluster 2, while Campsomerini mostly displayed as “-*trnM*-(-*trnL2*)” (mode 2) for cluster 1 and “*trnE-(-nad5)-(-trnH)*” (mode 4) for cluster 2. Surprisingly, *C. tartara* was always rearranged as the Scoliini referring above two clusters (mode 1 & 3). Within Scoliini, however, it exhibited fewer changes, with only one transposition of *trnE* in the *Scolia* sp. sequence. In Campsomerini, conversely, it still showed more changes though excluding shortly assembled *C. annulata* and unusual *C. tartara* referring tRNA cluster “*trnM*-*trnQ*”. *Phalerimeris phalerata*, exclusively, showed transpositions of *trnC*-*trnY* and *trnG*-*trnF* in Campsomerini or even in all Scoliidae. It coincided with the morphological appearance between these two tribes. Campsomerini, in general, display a more distinct sexual dimorphism and the genera are distinctive.

### 3.3. Phylogenetic Analyses

**Figure 5:**
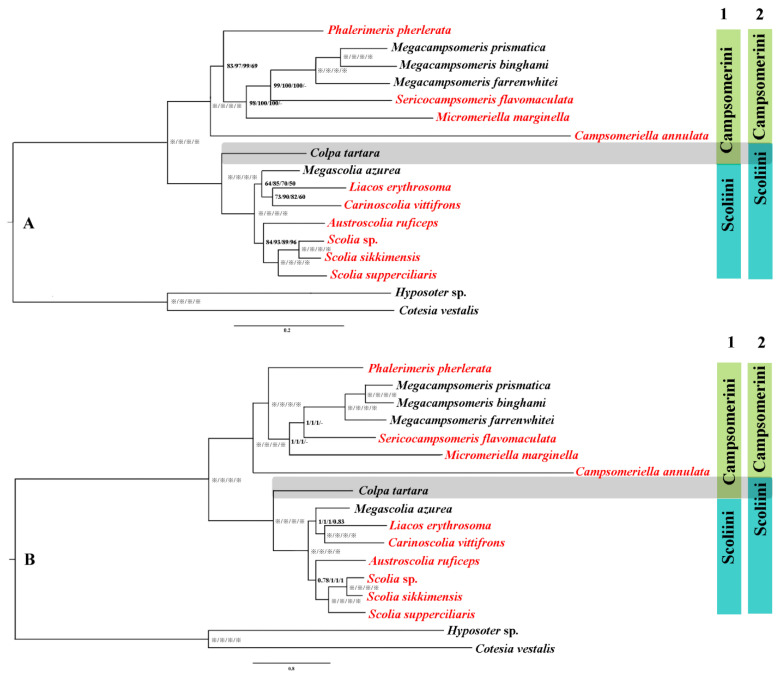
Phylogenetic tree of 15 representative lineages in Scoliidae based on matrices of PCG123/PCG123 + RNA/PCG12/AA separately. (**A**), the phylogenetic tree using maximum likelihood (ML) methods; (**B**), the phylogenetic tree using Bayesian inference (BI) methods. Note: “※” indicates bootstrap percentage = 100 or Bayesian posterior probability = 1; “-” means not applicable; 1 refers to former morphological attribution; 2 refers to new molecular attribution; red font refers to newly sequenced Scoliidae.

Phylogenetic relationships were inferred using MrBayes and IQtree based on four data matrices (PCG123, PCG123 + RNA, PCG12 and AA), which result in eight highly congruent tree topologies (Figure 5) with a high support value. Optimal partitioning strategy and models were selected using PartitionFinder v2.1.12 (Table S2). Generally, the support values of the corresponding nodes in the phylogenetic tree were relatively higher in BI than MI analysis in this study, which testified that MrBayes performs more reliably than ML methods in mitochondrial phylogenomic analysis of this group, corresponding with other Hymenoptera [14,52,53]. In all analyses, two clusters of the tribes Campsomerini and Scoliini were always recovered, which is consistent with former morphological [17,20] and *COI* analyses [1]. Within Campsomerini, three species of *Megacampsomeris* clustered together and they together were sister to *Sericocampsomeris* in BI and ML trees based on PCG123, PCG123 + RNA, PCG12 or (*Sericocampsomeris + Micromeriella*) based on AA matrixes (Figure 5). *Phalerimeris* together with the former genera was sister to *Campsomeriella* in all analyses. *Colpa tartara*, however, was always included as a sister group to other Scoliini members rather than clustered together with members of Campsomerini. Similar results were extracted when Yao et al. [33] used the same sequence to obtain the phylogenetic relationship of Scoliidae with other stinging wasps. Three *Scolia* species together with *Austroscolia* were sister to the cluster of *Megascolia +* (*Liacos* + *Carinoscolia*) in Scoliini in all analyses.

### 3.4. Distribution of Mitogenomic Rearrangements in Phylogenetic Trees of Scoliidae

**Figure 6:**
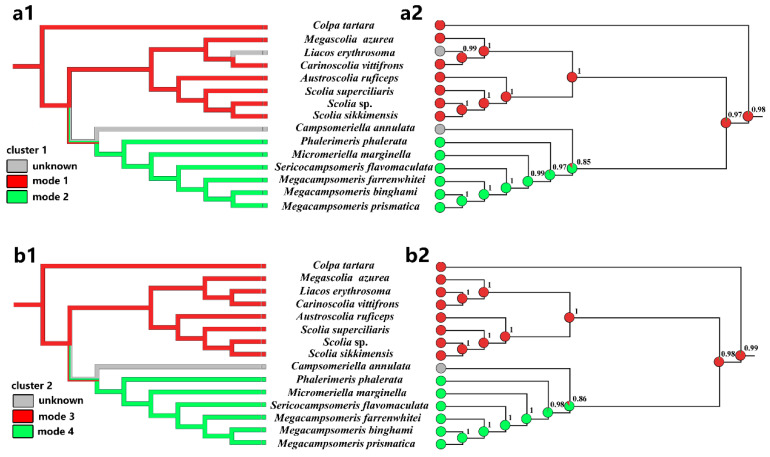
Reconstruction of ancestral states and mapping of mitogenomic clusters of different modes onto the Chinese Scoliidae tree (PCG123 + RNA). Two methods, maximum parsimony (**a1**,**b1**) and maximum likelihood (**a2**,**b2**) were applied for two clusters: cluster 1 (**a1**,**a2**) and cluster 2 (**b1**,**b2**). The area of colors at nodes in (**a2**,**b2**) corresponds to the probability of the given state, modes 1/2 and modes 3/4. The probability value for a more likely state was also given at these nodes. Mk1 model was applied for ML approach.

We considered PCG123 + RNA topology to analyze the clusters 1 and cluster 2 rearrangements in the phylogenetic context. Using these relationships, we mapped the mitogenomic features onto the topology and inferred ancestral states for the individual lineages using maximum parsimony (MP) and maximum likelihood (ML) methods (Figure 6).

Two methods applied to the topology for four modes in two clusters clearly indicated that the last common ancestor of Scoliidae contained a rearrangement of cluster 1 and 2 as the modes of Scoliini (modes 1 and 3) (Figure 6). This state was inherited also by the *Colpa* in its mitogenome, while Campsomerini excluding *Colpa* and unknown *Campsomeriella* rearranged a different mode referring cluster 1 and 2 (modes 2 and 4). The ML method provided the probability *p* = 0.977 (cluster 1)/0.987 (cluster 2) of this state for the last common ancestors of all Scoliidae. The last common ancestor of the Campsomerini groups could also rearrange as modes 1 (*p* = 0.849) & 3 (*p* = 0.860) as Scoliidae and so do the unknown modes of cluster 1 and 2 in *Campsomeriella annulata*. However, it will not happen in the last common ancestor of groups excluding *Campsomeriella* in Campsomerini (*p* = 0.966 (mode 2)/0.980 (mode 4)). In turn, an unknown rearrangement of cluster 1 in *Liacos erythrosoma* could be as Scoliini, with its last common ancestor rearranged as mode 1 with *p* = 0.988.

## 4. Discussion

### 4.1. Informative Mitochondrial Sequences and Phylogenetic Results

This is the first comprehensive study to use mitochondrial genomes to reconstruct the scoliid wasp phylogeny. In this study, 10 mitochondrial genomes representing eight genera of Scoliidae were newly sequenced. Comparative analysis including all Chinese Scoliidae genera provides valuable information for understanding the phylogenetic relationships within Scoliidae wasps. As members of the Hymenoptera, they showed high A + T compared to other orders in previous studies [40,53], while exhibiting lower content when compared to their “Parasitica” counterparts, which correspond to other Aculeata groups in Zheng et al. [11]. The results were similar to 13 protein-coding genes. However, we observed higher A + T for Scoliini than Campsomerini species except for *Colpa tartara*, which may suggest it is closer to Scoliini.

Gene rearrangements can help to define a variety of taxonomic scales below the ordinal level though no gene rearrangements are shared between insect orders [54,55]. In the Aculeata, gene rearrangement occurred in all species [11]. Synapomorphic rearrangement of rRNA genes and protein-coding genes were found in Scoliidae. The tRNA genes, however, had the highest frequency of rearrangement and were in sharp contrast between two tribes with the exception of *C. tartara* falling into the same mode of Scoliini in major rearrangements, which also suggested a closer relationship between *Colpa* and members of Scoliini rather than its traditional placement near the Campsomerini. We also found that *Campsomeriella annulata*, with the varied pattern gene rearrangement, was always inferred as being an outlier of Campsomerini and the longest branch in Scoliidae in later phylogenetic analysis. These indicated that an association between gene arrangement and sequence evolution may exist [11,56].

Phylogenetic relationships were inferred for all representative Chinese Scoliidae genera using MrBayes and IQtree based on four data matrices (PCG123, PCG123 + RNA, PCG12 and AA). The results in eight tree topologies showed high congruence with high support values excluding a slightly unstable position for (*Sericocampsomeris + Micromeriella*) in AA data matrices which may be the reason we lack sufficient representatives of these two groups. In general, MrBayes performs more reliably than ML methods in mitochondrial phylogenomic analysis of this group, corresponding with other Hymenoptera [14,52,53]. Our phylogenetic inferences support most of the relationships between genera and tribes of Scoliidae as in former morphological studies. *Colpa tartara*, however, was always included as a sister group to members of Scoliini, which is contrary to former morphological studies but consistent with recent studies of Khouri et al. [28] and Yao et al. [33].

We mapped the mitogenomic features onto the topology and inferred ancestral states for the individual lineages. Four mapped trees clearly indicated that the last common ancestor of Scoliidae contained a rearrangement of cluster 1 and 2 as the modes of Scoliini. This state was inherited also by the *Colpa* in its mitogenome, while other Campsomerini members rearranged more likely in a different mode, which is consistent with our other results for supporting *Colpa* as one of Scoliini.

### 4.2. Implications for Classification

In Scoliidae, the two *Proscolia* species distributed in Greece and Romania were believed to be the sister group to the remaining extant Scoliidae ever since they were described. And this is strongly supported by later authors [8,28,57]. For most of the remaining groups, on the contrary, the taxonomy has been historically unstable and confusing (see Elliott, 2011 [58]).

*Colpa*, containing about 29 species worldwide [2], is often mingled with other genera, especially *Trielis* and *Campsoscolia* (both synonymized later with *Colpa*) and its taxonomic treatment has historically varied significantly. Bradley [59] argued for a “basal” placement for *Campsoscolia*. Betrem [60] erected the tribe Trielini (emended by Betrem and Bradley [16] to Trielidini) within the Campsomerinae to contain the genera *Trielis* (treated as a subgenus of *Colpa*, except for *Guigliana*). For most of the following works, it was included as part of Campsomerinae or Campsomerini after being demoted by Rasnitsyn [17], except for Argaman [27] who erected a subfamily Colpinae for related species and placed it as a sister to his Scoliinae. Recently, more molecular evidence has been applied for supporting *Colpa* within the Scoliini clade [28,33] though lacking morphological discussion.

**Figure 7:**
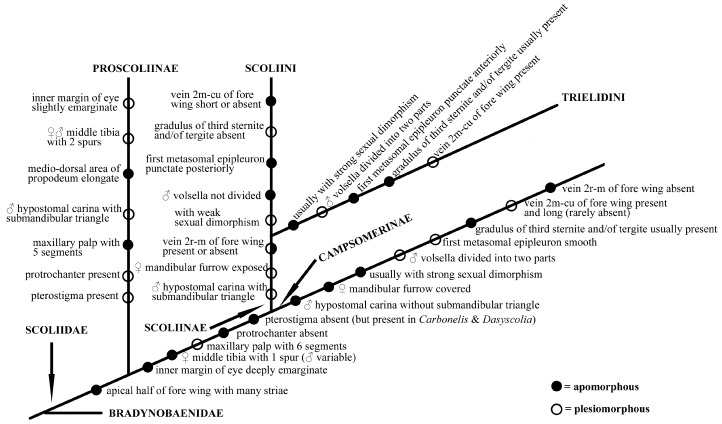
Hypothesis phylogram based on present study and former research on family Scoliidae.

*Colpa* was historically grouped in Campsomerini morphologically mainly for the presence of a vein 2m-cu of the fore wing which connected to the second submarginal cell and volsella of males divided into two parts [25]. Nevertheless, some differences can be observed between them, such as male hypostomal carina with submandibular triangle in the *Colpa* while being absent in Campsomerini, the female mandibular furrow being exposed while being concealed in Campsomerini, and the first metasomal epipleuron being punctate anteriorly but smooth in Campsomerini. Furthermore, the features mentioned above to distinguish *Colpa* from Campsomerini are all found in members of the tribe Scoliini, which justifies the inclusion of *Colpa* in the Scoliini considering the phylogenetic results and the similar AT content and gene rearrangements in our study; all analyses also strongly support *Colpa* as a member of Scoliini rather than Campsomerini. Hence, we propose a phylogram (Figure 7) hypothesis based on the present study, the suggestion by van Achterberg to treat the Campsomerini as subfamily and former research to explain the possible evolutionary directions of the family Scoliidae here. Nevertheless, more materials from other regions and related genera are needed to better understand the relationships between them.

## Figures and Tables

**Table 1:** Information on the 17 mitochondrial genomes of Hymenoptera used in this study.

Family	Tribe	Genus	Species	GenBank Accession No.	Reference
Braconidae		*Cotesia*	*Cotesia vestalis*	FJ154897	Wei et al. [42]
Ichneumonidae		*Hyposoter*	*Hyposoter* sp.	MG923499	Tang et al. [43]
Scoliidae (Scoliinae)	Campsomerini	*Campsomeriella*	*Campsomeriella annulata*	PP874260	This study
		*Colpa*	*Colpa tartara*	OM103697	Yao et al. [33]
		*Megacampsomeris*	*Megacampsomeris binghami*	OP946654	Huang et al. [14]
			*Megacampsomeris farrenwhitei*	OP946655	Huang et al. [14]
			*Megacampsomeris prismatica*	MH748671	Zheng et al. [11]
		*Micromeriella*	*Micromeriella marginella*	PP874264	This study
		*Phalerimeris*	*Phalerimeris phalerata*	PP874263	This study
		*Sericocampsomeris*	*Sericocampsomeris flavomaculata*	PP874266	This study
	Scoliini	*Austroscolia*	*Austroscolia ruficeps*	PP874258	This study
		*Carinoscolia*	*Carinoscolia vittifrons*	PP874259	This study
		*Liacos*	*Liacos erythrosoma*	PP874262	This study
		*Megascolia*	*Megascolia azurea*	OP661167	Liu et al. [32]
		*Scolia*	*Scolia* *sikkimensis*	PP874267	This study
			*Scolia superciliaris*	PP874265	This study
			*Scolia* sp.	PP874261	This study

## Data Availability

The data of the research were deposited in Hunan University of Arts and Science.

## Supplementary Materials

The following supporting information can be downloaded at: https://www.mdpi.com/article/10.3390/insects15100758/s1, Table S1: Detailed collecting data of the sequenced taxa.; Table S2: Opitimal partitioning schemes for mitogenome dataset determined by PartitionFinder.
