# Supplementary material for: Mitochondrial Phylogenomics of Scoliidae from China, with Evidence to Challenge the Former Placement of the Colpa Group
Source: Insects. 2024 Sep 30;15(10):758. doi: 10.3390/insects15100758 (PMC11508514; doi:10.3390/insects15100758)
Supplement: Supplementary file 1 [file insects-15-00758-s001.zip › insects-3190841-supplementary.pdf]

Table S1. Detailed collecting data of the sequenced taxa.

Species	Sex	Locality
Austroscolia ruficeps (Smith)	male	Guangdong, 23°9'20.7"N113°20'40.60"E
Carinoscolia vittifrons (Sichel)	male	Guangdong, 23°11'25.82"N113°21'47.01"E
Campsomeriella annulata (Fabricius, 1793)	male	Bhutan, 27°31'12.00"N89°52'12.00"E
Liacos erythrosoma (Burmeister)	female	Guangdong, 23°11'25.82"N113°21'47.01"E
Megacampsomeris binghami (Betrem)	male	Guangdong, 22°16'22.67"N111°11'38.7"E
Megacampsomeris farrenwhitei (Betrem)	male	Guangdong, 23°10'49.87"N113°21'21.33"E
Megascolia azurea (Christ)	male	Guangdong, 23°10'56.16"N113°21'28.94"E
Micromeriella marginella (Klug)	male	Hainan, 19°32'56.61"N110°47'28.82" E
Phalerimeris phalerata (de Saussure)	male	Guangdong, 23°10'49.87"N113°21'21.33"E
Scolia sikkimensis Bingham	male	Yunnan, 25°18'22.99"N98°47'37.34"E
Scolia superciliaris de Saussure	male	Guangdong, 23°10'49.87"N113°21'21.33"E
Scolia sp.	male	Shaanxi, 33°45'2.4"N108°41'32.09"E
Sericocampsomeris flavomacula Gupta & Jonathan	male	Hainan, 18°43'52"N110°11'30"E

Tables S2 Opitimal partitioning schemes for mitogenome dataset determined by PartitionFinder.

Dataset	Subset	Subset Partitions	Sites	Best Model
13PCG	1	n4p1, n1p1, a6p1, c3p1, cbp1, n3p1, n5p1, a8p2, c2p1, n4lp1	2683	GTR+G
	2	n4p2, n1p2, c3p2, c2p2, cbp2, a6p2, n5p2,	2523	GTR+G

		n3p2		
	3	n6p3, n2p3	582	GTR+G
	4	n5p3, n4p3, a6p3, n3p3, n1p3, a8p3, n4lp3	1830	GTR+G
	5	cbp3, c2p3, c3p3, c1p3	1371	GTR+G
	6	c1p1	518	GTR+G
	7	n2p1, n6p1, a8p1	638	GTR+G
	8	n6p2, n2p2, n4lp2	686	GTR+G
	9	c1p2	518	GTR+I+G
**PCG123**	1	c2p1, cbp1, c3p1	853	GTR+G
**+ RNA**	2	lr, n3p1, a8p2, n5p1, n4p1, a6p1, n1p1, sr, n4lp1	4614	GTR+I+G
	3	n4p2, n1p2, c3p2, c2p2, cbp2, a6p2, n3p2, n5p2	2523	GTR+G
	4	n1p3, n4lp3, a8p3, n5p3, n4p3, a6p3, n3p3	1830	GTR+G
	5	FcC_supermatrix, FcC_supermatrix	22698	GTR+I+G
	6	cbp3, c2p3, c3p3, c1p3	1371	GTR+G
	7	c1p2	518	GTR+I+G
	8	n2p1, a8p1, n6p1	638	GTR+G
	9	n2p3, n6p3	582	GTR+G
	10	n6p2, n2p2, n4lp2	686	GTR+G
	11	c1p1	518	GTR+G
**PCG12**	1	n6p2, n4lp2, n2p2	686	GTR+G
	2	n4p2, n1p2, c3p2, c2p2, cbp2, a6p2, n3p2, n5p2	2523	GTR+G
	3	n4p1, a6p1, n1p1, c3p1, cbp1, n3p1, a8p2, n5p1,	2683	GTR+G

		c2p1, n4lp1		
	4	c1p2	518	GTR+I+G
	5	n2p1, a8p1, n6p1	638	GTR+G
	6	c1p1	518	GTR+G
	7	FcC_supermatrix, FcC_supermatrix	22698	GTR+I+G
**AA**	1	n4l_aa_ali, a6_aa_ali, c3_aa_ali, cb_aa_ali, n4_aa_ali, n1_aa_ali, n5_aa_ali, c2_aa_ali, a8_aa_ali, n3_aa_ali, n2_aa_ali, n6_aa_ali	2704	MTART+I+G+F
	2	c1_aa_ali	515	MTART+G
